# Supplementary material for: Chromothripsis during telomere crisis is independent of NHEJ, and consistent with a replicative origin
Source: Genome Res. 2019 May;29(5):737–49. doi: 10.1101/gr.240705.118 (PMC6499312; doi:10.1101/gr.240705.118)
Supplement: Supplemental Material [file supp_gr.240705.118_Supplemental_file_1.zip › contigs/annotated_contigs/DB51/contig.2.DB51_length_682_mean_cov_6.03958944282.docx]

**DB51_length_682_mean_cov_6.03958944282**

```
TGAAGAAGGAGGATTGCTTGAGCTCAGGAGCTTAAGGTGGCAGTGAACTGTGATTGCATCACTGCACTCCAGCCTGGATGACAGCGTGA
 >chr15:25644502-25644833 - E=2e-184
GACCCTGTCTTTTAAAAACAAAAGAAACAAAAAAACAGACACATCGACCCACAAAACAGAATGGAGAGCACAGAAATAAATTCGTGTAC

TATCAGTTGATTTTGGACAGGGTGCAAAGAACACAGTGGGGAAAGGGAAGTCTGTTCAATAGGTGGTGTTGGGAAAATGGAATATCTAC

ATGCATGAGAATGAAATCAGACCTGTATCTCACATCATATACCAAATTCAAGTTAAAATGGATT|TATATATATATAAATAAAAGAATA
                                                                  >chr15:25643579-256439
AAATCAGTGCGCTGAAGAGATCTGTACTCCCATGTTCCTTTCGGTATCATTTGCAATACCCATGATACAGCATCAGCCTAAGTGTTCAT
30 - E=2e-199
GGATAAAGAAAATGTGGTATGTATACACAGTGGAATATACTTCAGCCATAAAAAGGAGGAAATGCTGTCATTTGTGACAGCAGGGATGA

ACCTGGAGGGCATTGTTGAAAGTAAGCCAGCCACAGAAAGGCAAATACTACATCTCTCTTAAATGTGGAATCTTAACTCAGGGATGTAA

AATGGTGGTTACCAGGGGCTGGGGGTGAGTGGACTGGGTTGCAGAGGTGTTGGTCAGAGG
```
